# What Is the Optimal Position of Low Tibial Tunnel in Transtibial Posterior Cruciate Ligament Reconstruction? A Quantitative Analysis Based on 2D CT Images and 3D Knee Models

**DOI:** 10.1111/os.14379

**Published:** 2025-02-26

**Authors:** Laiwei Guo, Xiaoyun Sheng, Caijuan Dai, Xingwen Wang, Lianggong Zhao, Xiaohui Zhang, Bin Geng, Zhongcheng Liu, Rui Bai, Xiaoli Zheng, Meng Wu, Yuanjun Teng, Yayi Xia

**Affiliations:** ^1^ Department of Orthopaedics Lanzhou University Second Hospital Lanzhou Gansu China; ^2^ Orthopaedics Clinical Medical Research Center of Gansu Province Lanzhou University Second Hospital Lanzhou Gansu China; ^3^ Intelligent Orthopaedic Industry Technology Center of Gansu Province Lanzhou University Second Hospital Lanzhou Gansu China; ^4^ Department of Pediatrics Lanzhou University Second Hospital Lanzhou Gansu China; ^5^ Department of Anesthesiology and Operation Lanzhou University Second Hospital Lanzhou Gansu China; ^6^ Academy for Engineering and Technology Fudan University Shanghai China; ^7^ Department of Orthopedic Surgery, Huashan Hospital Fudan University Shanghai China

**Keywords:** low tibial tunnel, position, posterior cruciate ligament, reconstruction

## Abstract

**Objectives:**

There is currently no consensus on the optimal placement of the low tibial tunnel for posterior cruciate ligament (PCL) reconstruction. This study aimed to perform the quantitative measurements of the optimal tangential low tibial‐tunnel (OTLT) parameters based on 2D CT images and 3D virtual knee models and expect to provide reference data for clinical creation of the OTLT during the arthroscopic transtibial PCL reconstruction.

**Methods:**

This was a retrospective CT image study. A total of 101 patients between January 2018 and December 2020 were included in our study for analysis. The CT image data of included patients were imported into Mimics software to create the 3D knee models, and the OTLT for PCL reconstruction was simulated on 2D CT images and 3D knee models, respectively. With that, the distances of the tunnel's entry (ADT) and exit points (BDT) to the tibial plateau, the length of the tunnel (LT), and the angle of the tunnel (AT) were measured. Variables were compared using the independent *t*‐test or the Mann–Whitney *u* test. Correlation analyses between the data and patient demographic factors were performed using the Pearson or Spearman correlation analysis. One‐way ANOVA was used to compare differences among height subgroups.

**Results:**

The mean ADT, LT, and AT on 2D CT images were 57.96 ± 5.34 mm, 39.92 ± 5.49 mm, and 37.23° ± 4.57° respectively, smaller than the values on 3D knee models (61.86 ± 6.80 mm, 45.56 ± 4.27 mm, and 48.17° ± 6.12°, all *p* values < 0.001). While the mean BDT on 2D CT images was significantly larger than 3D knee models (35.28 ± 3.07 mm vs. 29.72 ± 3.00 mm, *p* < 0.001). The BDT showed larger in males than females, the LT showed larger in the taller group, and the AT seemed to be larger in females and shorter people (all *p* values < 0.05).

**Conclusion:**

The quantitative parameters of the OTLT based on 2D CT images and 3D knee models can be used as reference data for clinical surgeons to build an anteromedial OTLT during the arthroscopic transtibial PCL reconstruction.

## Graphical abstract

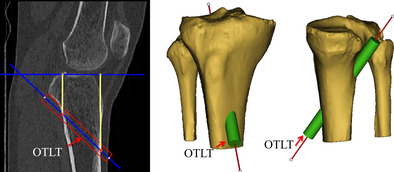

## 1 Introduction

Posterior cruciate ligament (PCL) reconstruction has been widely used as an important and effective treatment for patients with PCL injuries. Although previous studies have reported satisfactory clinical outcomes after PCL reconstruction, [1, 2, 3, 4, 5, 6, 7] the failure rate of PCL reconstruction is fairly high compared with anterior cruciate ligament (ACL) reconstruction [2, 3, 4, 5, 6, 7, 8]. The “killer turn” has been frequently documented as one of the primary reasons which will result in the graft failure [9, 10, 11]. Especially during the procedure of anatomic transtibial technique for PCL reconstruction, an acute angle inevitably forms at the proximal posterior tibia where the graft exits from the tibial tunnel [12]. This sharp angle of the graft at the tibial tunnel aperture creates a stress concentration point [13]. And it will gradually cause graft abrasion, thinning and permanent elongation, which might ultimately result in posterior laxity of the tibia and graft failure [14].

For PCL reconstruction, the most commonly used clinical method is anatomic transtibial technique [15]. However, this technique not only creates a sharp tibial tunnel edge but also may potentially damage the PCL remnants. The preservation of PCL remnant fibers is advantageous for maintaining proprioception, promoting graft revascularization and graft healing. Therefore, it is beneficial to make every effort to preserve these remnants during PCL reconstruction [1, 13, 16]. Moreover, several studies have demonstrated that the PCL tibial remnants worked as a soft tissue cushion, which could availably decrease the “killer‐turn” effect at the tunnel aperture [3, 17]. However, if the PCL remnants are kept in excess, difficulties may increase when the graft passes through the proximal tunnel aperture.

To reduce the killer turn and try the best to preserve the PCL remnants in the transtibial technique, researchers tend to modify placement of the tibial tunnel at the proximal posterior tibia. Okoroafor et al. [18] located the PCL non‐anatomic tibial tunnel at the proximal footprint of the PCL attachment. This technique might decrease the killer‐turn angle to a certain extent. However, it potentially receded the graft tension on the anterior–posterior orientation and consequently caused posterior tibial translation [18]. The other two researches Ahn [19] and Lee [16] located the tibial tunnel outlet at the distal lateral portion of the PCL tibial attachment within the anatomic PCL fossa (still above the so‐called “champagne glass drop‐off”) [20]. Alternatively, Fanelli [21, 22] recommended to create the tibial tunnel even lower at the inferior lateral part of the PCL fossa (just beneath the “champagne glass drop‐off”), based on the theory that this approach would break one acute angle down into two very smooth 45° angles at the proximal posterior tibia. Then the low tibial tunnel (Fanelli tunnel) technique can not only preserve more PCL remnants without additional difficulties in passing the graft but also effectively reduce the killer turn during transtibial PCL reconstruction. What's more, studies have shown excellent clinical and functional outcomes after long‐term follow‐up [20, 23]. And several authors reported that the low tibial tunnel group achieved equivalent or even more satisfactory outcomes compared with the anatomic tibial tunnel using the methods of biomechanics, finite element model, and 3D gait analysis [20, 24, 25, 26, 27]. Therefore, the low tibial tunnel placement is a fairly feasible clinical technique for transtibial PCL reconstruction.

Previous studies reported various locations of the low tibial tunnel and showed different results [20, 21, 24, 25, 26, 28]. While just like the research as Teng et al. [29] described, the tibial tunnel has a maximum allowable angle and the most appropriate position to reduce the killer turn for anatomic PCL reconstruction. When the angle of the low tibial tunnel was designed tangent to the posterior tibial cortex and the exit was just below the “champagne glass drop‐off,” it may reduce the killer turn to the greatest extent and avoid breaking down the posterior tibial wall [29]. We named it as the “optimal tangential low tibial‐tunnel” (OTLT) for transtibial PCL reconstruction. And to the best of our knowledge, quantitative analyses of the optimal location for low tibial tunnel in transtibial PCL reconstruction have not been reported so far.

The purposes of the present study were (1) to determine the OTLT location for the anteromedial approaches in transtibial PCL reconstruction, (2) to perform the quantitative measurements of OTLT parameters based on 2D CT images and 3D virtual knee models and compare the differences between groups, (3) to conduct the correlation analyses between the OTLT parameters and patient demographic factors (age, sex, height, and BMI).

## 2 Materials and Methods

### 2.1 Patients and Image Collection

After obtaining ethical approval from the ethics committee of our institution (No. 2022A‐556), we reviewed patients who underwent computed tomography (CT) scans between January 2018 and December 2020. The inclusion criteria were as follows: (1) the patient age was 18–60 years, (2) CT images showed high definition and standard, (3) the PCL tibial attachment could clearly show with the grayscale value manually adjusted, (4) Kellgren–Lawrence (K–L) scale less than 1 grade. Patients with any of the following conditions were excluded: (1) insufficient CT data, (2) a history of knee surgery, (3) concomitant fracture, tumor, deformity, and other pathological changes around the knee. Finally, 101 patients (48 males and 53 females) were enrolled in the study.

All the CT image data sets of included patients were collected. The CT scanner was 64‐multidetector‐row (Siemens AG) with a gantry rotation speed of 1.00 s/rotation, 0.625 mm collimation width × 12 detectors, a pitch factor of 0.90, and a field of view (FOV) of 25–30 cm. The images were saved in DICOM format.

### 2.2 Establishment of the OTLT on 2D CT Images and Quantitative Measurements

**FIGURE 1:**
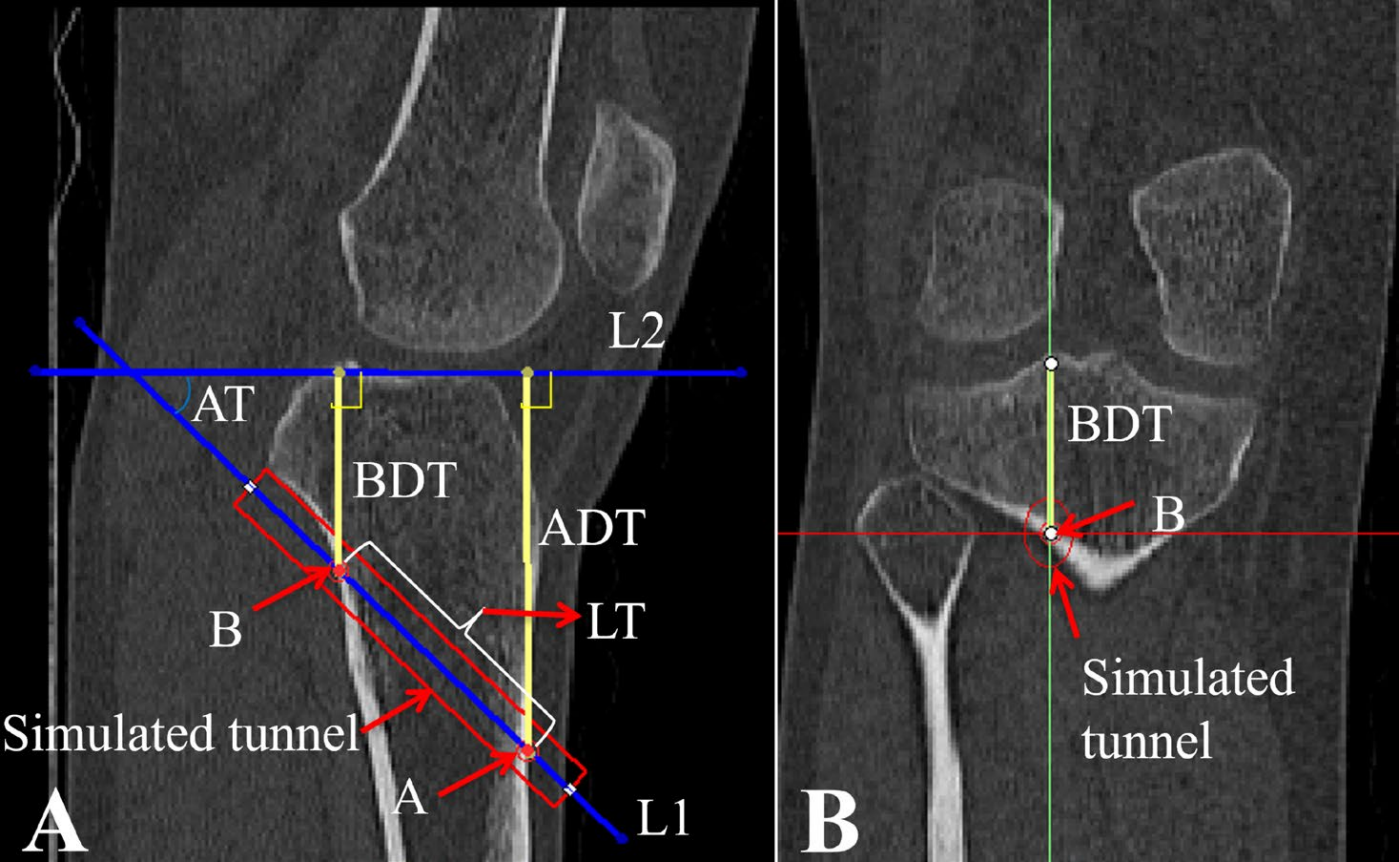
(A, B) Establishment of the optimal tangential low tibial‐tunnel (OTLT) on 2D CT images and quantitative measurements. (A) A 9 mm wide rectangle was built to simulate the tibial tunnel (red rectangle). Then the orientation of the simulated tunnel was adjusted so that the superior edge of the tunnel was tangent to the proximal posterior tibial cortex. (B) The coronal images were monitored to ensure the selected sagittal image slice through the lateral margin of the PCL anatomical tibial footprint. L1, the center line of the simulated tunnel; L2, the tibial plateau line; A, the tunnel's entry point; B, the tunnel's exit point; ADT, distance of the tunnel's entry point A to the tibial plateau; BDT, distance of the tunnel's exit point B to the tibial plateau; LT, length of the tunnel; AT, angle of the tunnel.

The obtained CT image data sets in DICOM format were imported into the Mimics software (Version 21.0, Materialise). With coronal images monitored, the sagittal slice through the lateral margin of the PCL anatomical tibial footprint was selected. Then the OTLT was simulated on 2D CT images using the following two steps. First, a 9 mm wide rectangle was built to simulate the tibial tunnel. Second, the orientation of the simulated tibial tunnel was adjusted so that the superior edge of the tunnel was tangent to the proximal posterior tibial cortex. Then a unique tibial tunnel was obtained (Figure 1).

After that, the relevant parameters were defined as follows: (1) the tunnel's entry point A was defined as the intersection point of the tunnel's center line L1 and the anterior tibial cortex; (2) the tunnel's exit point B was defined as the intersection point of the tunnel's center line L1 and the posterior tibial cortex; (3) the tibial plateau line L2 was defined as the tangential line passing through the anterior and posterior edges of the tibial plateau; (4) the distance of the tunnel's entry point to the tibial plateau (ADT) was defined as the perpendicular distance from point A to line L2; (5) the distance of the tunnel's exit point to the tibial plateau (BDT) was defined as the perpendicular distance from point B to line L2; (6) the length of the tunnel (LT) was defined as the distance from point A to point B; (7) the angle of the tunnel (AT) was defined as the angle formed by line L1 and line L2. Lastly, the quantitative values of ADT, BDT, LT, and AT were measured on the 2D CT images (Figure 1A).

### 2.3 Establishment of the OTLT on 3D Knee Models and Quantitative Measurements

**FIGURE 2:**
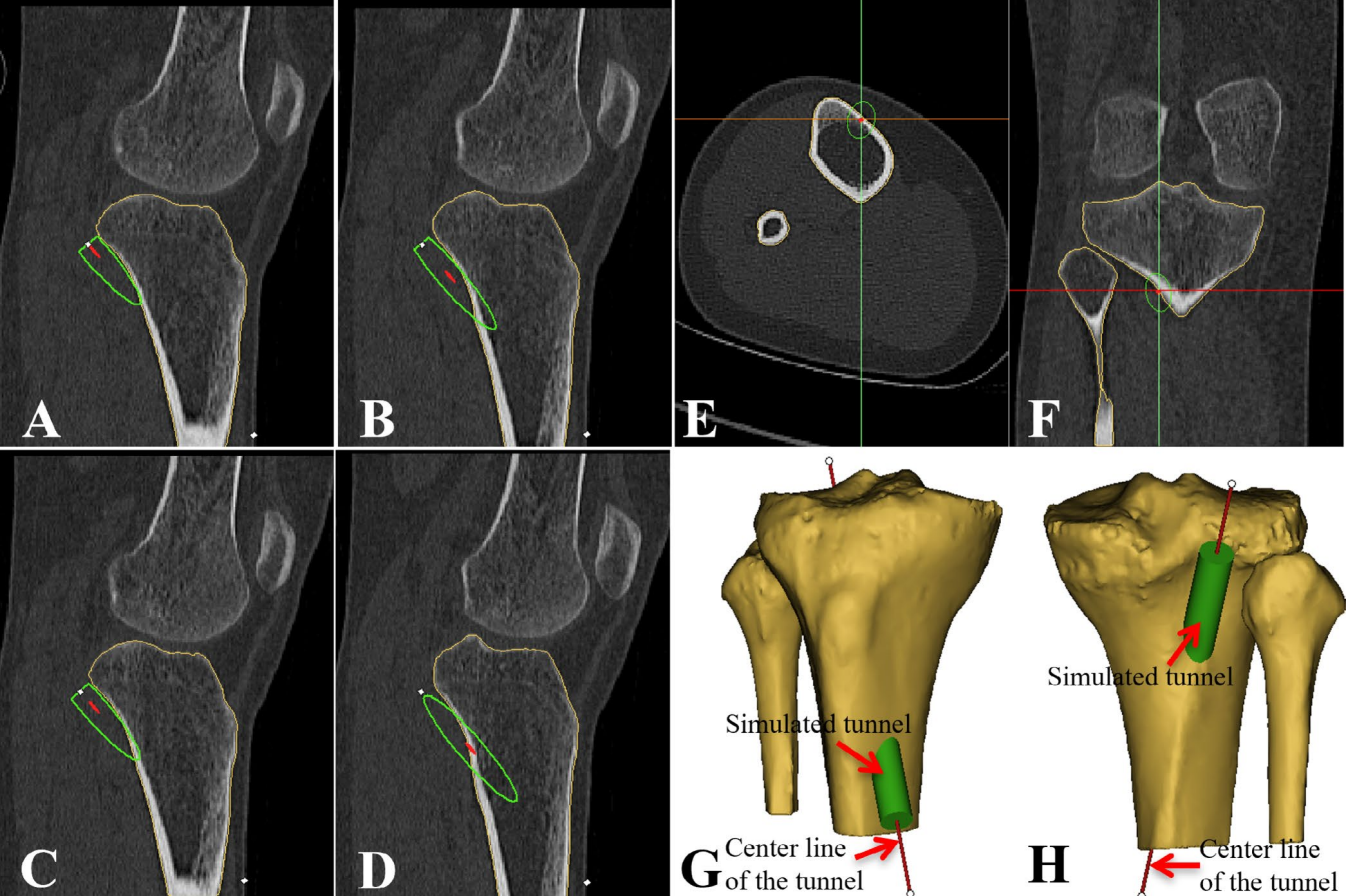
(A–H) Establishment of the optimal tangential low tibial‐tunnel (OTLT) on 3D knee models. (A–D) With the outline of the simulated tunnel was monitored on successive 2D sagittal planes, the orientation of the simulated tunnel was constantly adjusted so that the superior edge of the tunnel was tangent to the posterior tibial cortex. (E) The axial images were monitored to ensure the tunnel's entry point was located on the anterior tibial cortex 15 mm medial to the tibial crest. (F) The coronal images were monitored to ensure the tunnel's exit point was located on the line through the lateral margin of the PCL anatomical tibial footprint. (G, H) The simulated tunnel was monitored on 3D model to ensure that no fracture and interstice occurred at the proximal posterior tibial cortex (G, anterior view; H, posterior view).

**FIGURE 3:**
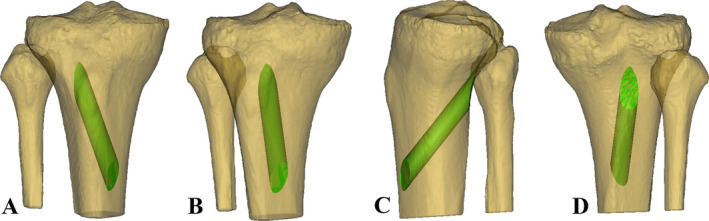
The green overlap region between the cylinder and 3D tibia was exactly the “optimal tangential low tibial‐tunnel” (OTLT) ((A) anterior view; (B) anteromedial view; (C) lateral view; (D) posterior view).

The 2D CT image data sets were composited and reconstructed into a 3D knee model via Mimics software. Then a virtual computer simulation of the 3D OTLT reconstruction was performed on the model. First, a 9 mm diameter cylinder passing through the proximal tibia was created to simulate the PCL tibial tunnel. Second, with successive 2D CT sagittal images monitored, the orientation of the simulated tunnel on 3D model was constantly adjusted so that the superior edge of the tunnel was as close as possible, equivalently tangent to the proximal posterior tibial cortex (Figure 2A–D). Third, the tunnel was fine‐tuned to ensure the tunnel's entry point was located on the anterior tibial cortex 15 mm medial to the tibial crest, and the tunnel's exit point was located on the line through the lateral margin of the PCL anatomical tibial footprint on the axial and coronal images, respectively (Figure 2E,F). Throughout the process, the second and third steps may need to be repeated several times to acquire the ultimately optimal position of the tunnel, and simultaneously, the 3D model will be also monitored to ensure that no fracture and interstice occurred at the proximal posterior tibial cortex (Figure 2G,H). Finally, an eligible cylindrical tunnel was obtained, and the overlap region of the cylinder and 3D tibia was exactly the OTLT we wanted (Figure 3).

**FIGURE 4:**
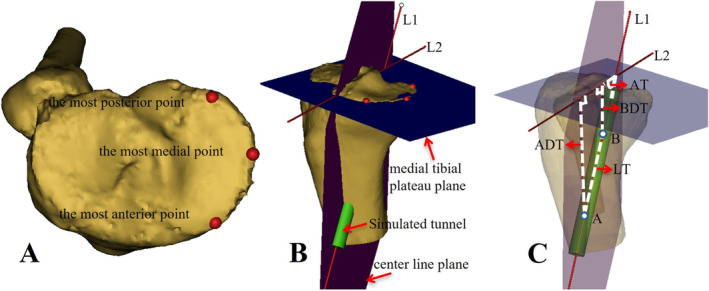
(A–C) Quantitative measurements of the optimal tangential low tibial‐tunnel (OTLT) on 3D knee models. (A, B) The reference plane of the medial tibial plateau was created using the most anterior, posterior, and medial points on the medial tibial plateau. Then another plane passing through the tunnel's center line and perpendicular to the medial tibial plateau plane was created. The tibial plateau line was defined as the intersecting line of the two reference planes. (C) Parameter values of the OTLT were measured. L1, the center line of the simulated tunnel; L2, the tibial plateau line; A, the tunnel's entry point; B, the tunnel's exit point; ADT, distance of the tunnel's entry point A to the tibial plateau; BDT, distance of the tunnel's exit point B to the tibial plateau; LT, length of the tunnel; AT, angle of the tunnel.

We defined the medial tibial plateau as the reference plane for subsequent measurements according to a previous study [29]. And the reference plane of the medial tibial plateau was created using three points: the most anterior, posterior, and medial points on the medial tibial plateau [30] (Figure 4A,B). Then we created another plane passing through the tunnel's center line L1 and perpendicular to the medial tibial plateau plane. The tibial plateau line L2 was defined as the intersecting line of the two reference planes (Figure 4B). The tunnel's entry point A and exit point B were respectively defined as the intersection points of the tunnel's center line and the tibial cortex. Lastly, just like on 2D CT images, the parameters of ADT, BDT, LT, and AT were defined and measured on the 3D knee models (Figure 4C).

### 2.4 Reliability Evaluating

Intraclass correlation coefficients (ICCs) were used to evaluate measurement reliability according to the following procedures: One senior orthopedic surgeon performed the measurements of all parameters twice at a 4‐week interval to assess the intraobserver reliability. Another orthopedic surgeon independently measured the parameters to perform the assessment of interobserver reliability. An ICC less than 0.40 was considered as poor reliability, whereas ICCs of 0.40–0.59, 0.60–0.74, and 0.75–1.00 were respectively considered as fair, good, and excellent reliability [31]. Finally, all ICCs for measurement results were > 0.75 (range 0.85–0.97), indicating satisfactory intra‐ and inter‐observer reliabilities.

### 2.5 Statistical Analysis

The sample size calculation was performed using G*Power (Version3.1.9; Heinrich Heine University, Dusseldorf) based on preliminary measurement data obtained previously. We estimated that with a sample size of 90 patients, the study would have more than 90% power to detect a between‐group difference based on the effect size of 0.40 and a two‐sided *t*‐test with a significance level of 0.05.

Statistical analysis was performed using SPSS (Version 25.0; IBM Corp.). All data were identified as continuous quantitative variables. Kolmogorov–Smirnov test was used to determine whether the quantitative variables of each group obeyed normal distribution. Normally distributed quantitative data were presented as the mean and standard deviation (height, weight, body mass index, ADT, BDT, LT, AT), and non‐normally distributed data were presented as median and range (age). Quantitative variables were compared using the independent *t*‐test for normally distributed continuous variables or the Mann–Whitney *u* test for non‐normally distributed data. The mean differences with 95% confidence intervals for continuous quantitative variables were calculated. To analyze the correlation between the data and patient demographic factors, the Pearson correlation analysis was performed for normally distributed variables (height, weight, body mass index), while Spearman correlation analysis for non‐normally distributed data (age, gender). A correlation coefficient of < 0.3, 0.3–0.7, and > 0.7 were considered as weak, moderate, and strong correlation, respectively. After that, subgroup analyses based on gender and height factors were conducted to compare the differences in outcomes. One‐way ANOVA was used to compare differences among height groups. A *p* value < 0.05 was considered statistically significant.

## 3 Results

### 3.1 Patient Demographic Data

Based on the inclusion and exclusion criteria, 101 patients were included in our study for analysis. There were 48 males and 53 females, the proportions of the left and right knees were basically the same, the median age of the patients was 36 years (range: 18–60 years), and the mean height, weight and body mass index (BMI) were 1.67 ± 0.08 m, 64.63 ± 11.20 kg, and 23.17 ± 3.37 kg/m^2^, respectively. The detailed characteristics of patients are included in Table 1.

**TABLE 1:** Patient demographic data (*n* = 101)^a^.

Demographic factor	Value
Male	48 (47.5%)
Female	53 (52.5%)
Left knee	50 (49.5%)
Right knee	51 (50.5%)
Age, years	36 (18–60)
Height, m	1.67 ± 0.08
Weight, kg	64.63 ± 11.20
BMI, kg/m^2^	23.17 ± 3.37

Abbreviation: BMI, body mass index.

^a^ Data are presented as numbers (percentage), median (range), or mean ± standard deviation.

### 3.2 The ADT, BDT, LT, and AT of the OTLT on 2D CT Images and 3D Knee Models

The mean ADT, LT, and AT relative to the tibial plateau on 2D CT images were 57.96 ± 5.34 mm, 39.92 ± 5.49 mm, and 37.23° ± 4.57°, respectively, which were significantly less than the values on 3D knee models (61.86 ± 6.80 mm, 45.56 ± 4.27 mm, and 48.17° ± 6.12°), with the mean differences of −3.90 mm (95% CI: −5.59 to 2.20 mm, *p* < 0.001), −5.65 mm (95% CI: −7.01 to −4.28 mm, *p* < 0.001) and − 10.94° (95% CI: −12.44° to −9.44°, *p* < 0.001). While the mean BDT on 2D CT images was significantly larger than 3D knee models (35.28 ± 3.07 mm vs. 29.72 ± 3.00 mm), with the mean difference of 5.56 mm (95% CI: 4.72 to 6.40, *p* < 0.001) (Table 2).

**TABLE 2:** Measurements of ADT, BDT, LT, and AT on 2D CT images and 3D knee models (*n* = 101)^a^.

Parameter	Mean ± SD		Mean difference (95% CI)	*t*	*p*
	2D	3D			
ADT, mm	57.96 ± 5.34	61.86 ± 6.80	−3.90 (−5.59 to 2.20)	−4.529	0.000
BDT, mm	35.28 ± 3.07	29.72 ± 3.00	5.56 (4.72 to 6.40)	13.018	0.000
LT, mm	39.92 ± 5.49	45.56 ± 4.27	−5.65 (−7.01 to −4.28)	−8.155	0.000
AT, degree	37.23 ± 4.57	48.17 ± 6.12	−10.94 (−12.44 to −9.44)	−14.397	0.000

Abbreviations: 2D, two‐dimension; 3D, three‐dimension; AT, angle of the tunnel; CI, confidence interval; LT, length of the tunnel; SD, standard deviation.

^a^ ADT, distance of the tunnel's entry point A to the tibial plateau; BDT, distance of the tunnel's exit point B to the tibial plateau.

### 3.3 Correlation Analyses Between the OTLT Parameters and Patient Demographic Data

Correlation analyses between outcomes and patient demographic factors are shown in Table 3. The AT on 3D knee models had a moderate positive correlation with gender (correlation coefficient = 0.468, *p* < 0.001), while the ADT, BDT, AT on 2D CT images and the BDT on 3D knee models showed weak correlations (correlation coefficients: −0.209, *p* = 0.036; −0.288, *p* = 0.004; 0.204, *p* = 0.041 and − 0.222, *p* = 0.026, respectively). The LT on 2D CT images and 3D knee models both showed moderate positive correlations with height (correlation coefficients: 0.352, *p* < 0.001 and 0.318, *p* = 0.001, respectively). The ADT on 2D CT images and the BDT, AT on 3D knee models showed weak correlations with height (correlation coefficients: 0.245, *p* = 0.014; 0.245, *p* = 0.014 and − 0.291, *p* = 0.003 respectively). The BDT, LT, and AT on 2D CT images showed weak correlations with age (correlation coefficients: −0.283, *p* = 0.004; −0.197, *p* = 0.048 and 0.199, *p* = 0.046, respectively), and the AT on 3D knee models had a weak correlation with weight (correlation coefficient: −0.250, *p* = 0.012). All parameters on 2D CT images and 3D knee models showed no correlations with BMI (*p* > 0.05).

**TABLE 3:** Correlation analyses between outcomes and patient demographic factors^a^.

Parameter	Gender		Age		Height		Weight		BMI	
	*r* ^b^	*p*	*r* ^b^	*p*	*r* ^c^	*p*	*r* ^c^	*p*	*r* ^c^	*p*
2D‐ADT, mm	−0.209*	0.036	−0.171	0.087	0.245*	0.014	0.050	0.620	−0.104	0.302
2D‐BDT, mm	−0.288**	0.004	−0.283**	0.004	0.168	0.094	0.049	0.624	−0.046	0.650
2D‐LT, mm	−0.098	0.332	−0.197*	0.048	0.352**	0.000	0.147	0.143	−0.058	0.564
2D‐AT, degree	0.204*	0.041	0.199*	0.046	−0.166	0.098	−0.111	0.271	−0.029	0.771
3D‐ADT, mm	0.143	0.154	−0.084	0.403	0.112	0.267	−0.070	0.487	−0.156	0.120
3D‐BDT, mm	−0.222*	0.026	0.007	0.944	0.245*	0.014	0.008	0.938	−0.145	0.149
3D‐LT, mm	−0.167	0.095	−0.010	0.918	0.318**	0.001	0.139	0.166	−0.044	0.661
3D‐AT, degree	0.468**	0.000	−0.037	0.715	−0.291**	0.003	−0.250*	0.012	−0.129	0.197

Abbreviations: 2D, two‐dimension; 3D, three‐dimension; AT, angle of the tunnel; BMI, body mass index; LT, length of the tunnel.

^a^ ADT, distance of the tunnel's entry point A to the tibial plateau; BDT, distance of the tunnel's exit point B to the tibial plateau.

^b^ *r*, Spearman correlation coefficient.

^c^ *r*, Pearson correlation coefficient.

* Showed significant correlation at the level of 0.05 (two‐sided).

** Showed significant correlation at the level of 0.01 (two‐sided).

### 3.4 Subgroup Analysis

Based on the results of correlation analyses, we conducted subgroup analyses for gender and height. Gender subgroup analysis showed that the mean ADT, BDT on 2D CT images and BDT on 3D knee models were larger in males than females (59.16 ± 5.33 mm vs. 56.88 ± 5.15 mm; 36.26 ± 3.17 mm vs. 34.38 ± 2.70 mm and 30.43 ± 3.06 mm vs. 29.07 ± 2.82 mm, respectively), with the mean differences of 2.29 mm (95% CI: 0.22 to 4.36 mm, *p* = 0.031), 1.88 mm (95% CI: 0.72 to 3.04 mm, *p* = 0.002), and 1.36 mm (95% CI: 0.20 to 2.52 mm, *p* = 0.022). While the mean AT on 3D knee models was significantly smaller in males than females (45.17° ± 5.43° vs. 50.89° ± 5.43°), with the mean difference of −5.72° (95% CI: −7.86° to −3.57°, p < 0.001). The LT, AT on 2D CT images and ADT, LT on 3D knee models showed no significant differences between males and females (*p* > 0.05) (Table 4).

**TABLE 4:** Comparison between subgroups based on gender^a^.

Parameter	Mean ± SD		Mean difference (95% CI)	*t*	*p*
	Male (*n* = 48)	Female (*n* = 53)			
2D‐ADT, mm	59.16 ± 5.33	56.88 ± 5.15	2.29 (0.22 to 4.36)	2.192	0.031
2D‐BDT, mm	36.26 ± 3.17	34.38 ± 2.70	1.88 (0.72 to 3.04)	3.212	0.002
2D‐LT, mm	40.63 ± 5.46	39.28 ± 5.50	1.35 (−0.82 to 3.52)	1.237	0.219
2D‐AT, degree	36.30 ± 4.25	38.08 ± 4.73	−1.78 (−3.56 to 0.004)	−1.980	0.051
3D‐ADT, mm	60.86 ± 5.68	62.76 ± 7.62	−1.90 (−4.57 to 0.78)	−1.406	0.163
3D‐BDT, mm	30.43 ± 3.06	29.07 ± 2.82	1.36 (0.20 to 2.52)	2.321	0.022
3D‐LT, mm	46.27 ± 4.00	44.92 ± 4.44	1.35 (−0.33 to 3.02)	1.598	0.113
3D‐AT, degree	45.17 ± 5.43	50.89 ± 5.43	−5.72 (−7.86 to −3.57)	−5.285	0.000

Abbreviations: 2D, two‐dimension; 3D, three‐dimension; AT, angle of the tunnel; CI, confidence interval; LT, length of the tunnel; SD, standard deviation.

^a^ ADT, distance of the tunnel's entry point A to the tibial plateau; BDT, distance of the tunnel's exit point B to the tibial plateau.

Height subgroup analysis showed that the mean ADT and LT were both larger in group of ≥ 1.71 m compared with the groups of ≤ 1.60 m and 1.61–1.70 m on 2D CT images (ADT: 60.51 ± 4.57 mm vs. 56.64 ± 4.63 mm and 57.23 ± 5.67 mm; LT: 42.68 ± 4.89 mm vs. 37.50 ± 5.58 mm and 39.63 ± 5.19 mm, intragroup *p* value < 0.05). The mean AT on 3D knee models was smaller in group of ≥ 1.71 m compared with the groups of ≤ 1.60 m and 1.61–1.70 m (44.96° ± 5.99° vs. 50.12° ± 4.83° and 48.95° ± 6.20°, intragroup *p* value < 0.05). The mean BDT on 3D knee models was larger in group of ≥ 1.71 m compared with the groups of ≤ 1.60 m (30.88 ± 3.30 mm vs. 28.77 ± 2.58 mm, intragroup *p* value < 0.05). The mean values of LT in groups of 1.61–1.70 m and ≥ 1.71 m were larger than in group of ≤ 1.60 m on 3D knee models (45.64 ± 4.39 mm and 47.30 ± 3.84 mm vs. 43.55 ± 3.72 mm, intragroup *p* value < 0.05). The BDT, AT on 2D CT images and ADT on 3D knee models showed no significant differences among groups (intergroup *p* value > 0.05) (Table 5).

**TABLE 5:** Comparison among subgroups based on height^a^.

Parameter	Mean ± SD			*F*	*p* (intergroup)
	≤ 1.60 m (*n* = 25)	1.61–1.70 m (*n* = 49)	≥ 1.71 m (*n* = 27)		
2D‐ADT, mm	56.64 ± 4.63	57.23 ± 5.67	60.51 ± 4.57* ^,^ **	4.603	0.012
2D‐BDT, mm	34.79 ± 2.34	35.02 ± 3.46	36.19 ± 2.80	1.694	0.189
2D‐LT, mm	37.50 ± 5.58	39.63 ± 5.19	42.68 ± 4.89* ^,^ **	6.557	0.002
2D‐AT, degree	38.00 ± 5.45	36.91 ± 4.59	37.09 ± 3.65	0.481	0.620
3D‐ADT, mm	60.23 ± 5.48	62.99 ± 7.74	61.32 ± 5.85	1.490	0.231
3D‐BDT, mm	28.77 ± 2.58	29.55 ± 2.87	30.88 ± 3.30*	3.533	0.033
3D‐LT, mm	43.55 ± 3.72	45.64 ± 4.39*	47.30 ± 3.84*	5.470	0.006
3D‐AT, degree	50.12 ± 4.83	48.95 ± 6.20	44.96 ± 5.99* ^,^ **	5.933	0.004

Abbreviations: 2D, two‐dimension; 3D, three‐dimension; AT, angle of the tunnel; LT, length of the tunnel; SD, standard deviation.

^a^ ADT, distance of the tunnel's entry point A to the tibial plateau; BDT, distance of the tunnel's exit point B to the tibial plateau.

* The *p* value < 0.05 compared with the group of ≤ 1.60 m.

** The *p* value < 0.05 compared with the group of 1.61–1.70 m.

## 4 Discussion

The principal finding of this study was that we quantitatively determined the parameter values of the OTLT based on 2D CT images and 3D knee models. They can be used as reference data for clinical surgeons to build an anteromedial OTLT during the arthroscopic transtibial PCL reconstruction.

To reduce the “killer turn” effect and pursue more excellent clinical and functional outcomes, quite a few researchers attempted to conduct a low tibial tunnel during arthroscopic PCL reconstruction [20, 21, 24, 25, 26]. However, at present little is known about how to perform an optimal placement of the low tibial tunnel and the quantitative parameter values of the accurate location are deficient. To our knowledge, this is the first research to simulate the optimal location of the low tibial tunnel and conduct quantitative measurements of relevant parameters on 2D CT images and 3D knee models. These quantitative parameter values would provide reference data for orthopedic surgeons to create an optimal low tibial tunnel during arthroscopic transtibial PCL reconstruction.

### 4.1 Determination of the OTLT Location

Previous studies have described the technique of low tibial tunnel. Fanelli et al. [21] proposed that the outlet of the tibial tunnel was located at the inferior lateral aspect of the PCL anatomic insertion site. Yoon et al. [20] located the target point of the tibial tunnel 5–10 mm below the “champagne glass drop‐off,” or lateral and distal of the PCL fossa. And Lin et al. [25] drilled the guide pin from 40 to 50 mm below the tibial plateau with an angle of 60° to the point of 15–18 mm distal and slightly lateral to the center of the PCL footprint. However, no consensus has been reached regarding the accurate and optimal position of the low tibial tunnel and no quantitative measurements of relevant parameters were conducted.

**FIGURE 5:**
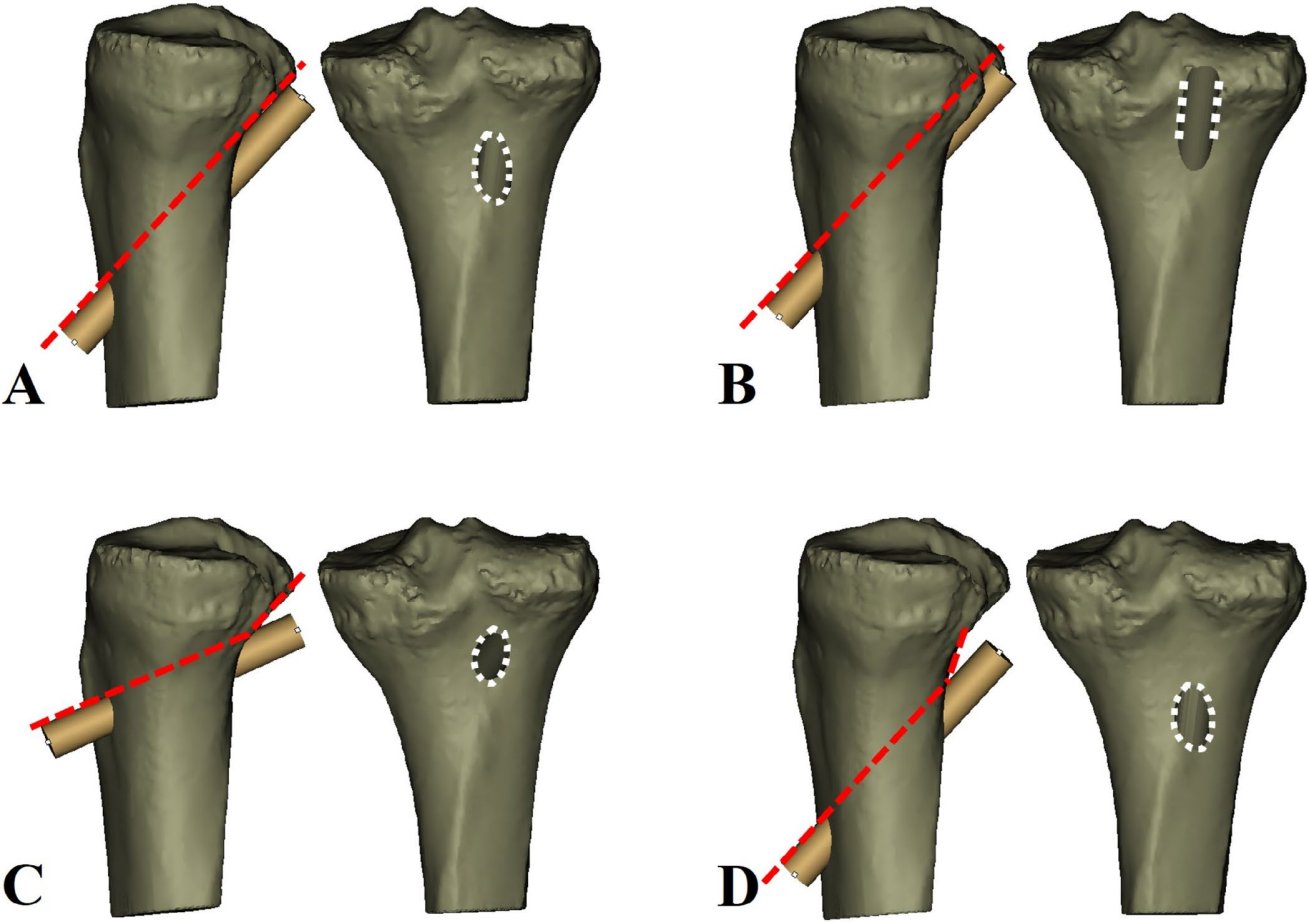
(A–D) Outcomes of different tunnel positions. (A) The optimal tangential low tibial‐tunnel (OTLT) was tangent to the proximal posterior tibial wall. (B) The tibial tunnel was located higher resulting that the posterior tibial wall was broken down and a rough groove with sharp edges was produced. (C, D) The tibial tunnel was located lower (D) or non‐tangential to the proximal posterior tibial cortex (C) resulting in an additional “kill‐turn” between the posterior tibial cortex and the proximal aperture of the tunnel.

Based on that, our study used the 2D CT images and 3D knee models to simulate the low tibial tunnel for PCL reconstruction. We created a simulated tunnel just below the “champagne glass drop‐off” [20] and adjusted the direction of the tunnel to make it as close to the proximal posterior tibial cortex as possible, that is to say, make the superior wall of the tunnel tangent to the posterior cortex (Figure 5A). We simultaneously ensured the tunnel's entry point was located on the anterior tibial cortex 15 mm medial to the tibial crest, and the tunnel's exit point was located on the line through the lateral margin of the PCL anatomical tibial footprint (Figure 2E,F). After that, a unique tibial tunnel was obtained.

If the tunnel was located higher, lower, or non‐tangential to the proximal posterior tibial cortex, two outcomes were produced. One was that the posterior tibial wall would be broken down and a rough groove with sharp edges was produced, resulting in the aggravation of abrasion when the graft passed through the groove (Figure 5B). The other was that an additional “kill‐turn” would be created between the posterior tibial cortex and the proximal aperture of the tunnel, similarly leading to excessive graft wear and increased risks of iatrogenic popliteal neurovascular injury (Figure 5C,D). While the aforementioned position of the unique tunnel may be the most appropriate to effectively avoid the two outcomes. Therefore, we defined the unique target tunnel as the “optimal tangential low tibial‐tunnel” (OTLT) for transtibial PCL reconstruction.

### 4.2 Quantitative Parameter Values of the OTLT on 2D CT Images and 3D Knee Models

The parameters of ADT, BDT, LT, and AT were measured on 2D CT images and 3D knee models. On 3D knee models, the mean ADT and BDT were about 62 mm and 30 mm, respectively; the mean LT and AT were about 46 mm and 48°, respectively. That means when we plan to build an OTLT for arthroscopic transtibial PCL reconstruction, we need to locate the PCL drilling guide pin from 62 mm below the tibial plateau and 15 mm medial to the tibial crest with an angle of 48°, toward the exit point 30 mm below the tibial plateau and inferior lateral aspect of the PCL anatomic insertion site. In addition, we should control the depth of 46 mm (41–50 mm) when drilling the guide pin and the reamer to reduce the risks of neurovascular injury.

With regard to the location of the tunnel's exit point on the proximal‐to‐distal direction, Yoon et al. [20] tended to locate it at the position 5–10 mm below the “champagne glass drop‐off”, and Lin et al. [25] suggested the point 15–18 mm distal to the center of the PCL footprint. While our study showed that the distance of the tunnel's exit point was about 30 mm referring to the tibial plateau, which the author thought was more feasible to determine the insertion depth of the drilling guide arm during arthroscopic transtibial PCL reconstruction. In addition, another study by our team showed the perpendicular distance of the PCL anatomic insertion relative to the tibial plateau was about 15 mm based on a large sample MRI analysis [32]. In combination with the value of BDT in the current study, it gives us a warning to some extent that if the drilling guide arm is inserted into the depth between 15 mm and 30 mm during PCL reconstruction, the proximal posterior tibial wall may probably be broken down and resulting in a rough groove with sharp edges as mentioned above (Figure 5B). Even though the methods of the two studies were different, the authors believed that this information was still a valuable reference for surgeons.

On 2D CT images, the mean ADT, BDT, LT, and AT were about 58 mm, 35 mm, 40 mm and 37°, respectively. These values can assist us to draw up a plan about the distances (ADT, BDT), depth (LT), and angle (AT) of the OTLT on preoperative CT images. However, we noticed that the values of ADT, LT, and AT were smaller, except the BDT, on 2D CT images than 3D knee models. The mean differences of ADT, BDT, LT, and AT were about −4 mm, 6 mm, −6 mm, and − 11°, respectively. The reasons for these differences might be that the tunnel entrance could not be adjusted from lateral to medial via a 2D image. If the OTLT was clinically planned on a 2D sagittal CT image, the tunnel entrance would be estimated approximately on the tibial crest. This was not in accordance with clinical practice because few surgeons used the tibial crest approach for PCL reconstruction. As 3D image simulation technology has been proved to provide an excellent 3D perspective of bone morphology and show high accuracy and reliability for simulated tunnel location and measurements [33, 34], it is more consistent with the actual surgical approaches of PCL reconstruction when making plans on 3D knee models. Therefore, surgeons should be aware that the preoperative plans for an OTLT on 2D CT image may not represent the actual parameter values during PCL reconstruction procedure. The differences may need to be added on for more accurate location in practical surgery operations.

### 4.3 Correlation Analyses Between the OTLT Parameters and Patient Demographic Factors

Our results showed some parameters had significant correlations with the gender and height factors, but they had no obvious correlations with age, weight, and BMI. Subgroup analyses for gender and height were significant for individual customization of the OTLT for different populations. The BDT showed larger in males than females, the LT showed larger in the taller group, while the AT seemed to be larger in females and shorter people. This is important information because the BDT, AT, and LT were vital parameters for locating the tibial tunnel during arthroscopic PCL reconstruction. The BDT indicated the insertion depth of the PCL drilling guide arm at the posterior inferior aspect of the tibial plateau, the AT ensured the tunnel tangent to the posterior tibial wall and the LT contributed to restricting the depth of the reamer in surgery. Therefore, surgeons should moderately adjust the BDT, AT, and LT values of OTLT during PCL reconstruction according to gender and height, in case of abnormal tunnel position, posterior tibial wall fractures, and neurovascular injuries.

### 4.4 Strengths and Limitations

This study innovatively proposed the OTLT and conducted quantitative measurements of relevant parameters based on 2D CT images and 3D knee models. These quantitative parameter values could be used as reference data for orthopedic surgeons to accurately create an OTLT during arthroscopic transtibial PCL reconstruction. This study has several potential limitations. First, MRI examination is the most widely used technique for PCL injury and it would be more beneficial for preoperative planning. As soft tissue and cartilage on CT images may not be clear enough, some measuring deviations might exist. However, it was unavailable to produce an accurate 3D knee model on MRI series. We created 3D knee models to simulate the tunnel and performed the measurements based on CT images. Second, our study only simulated the anteromedial OTLT without an anterolateral approach. Several studies reported that the anterolateral approach might reduce the killer turn on the coronal plane than anteromedial approach [35]. However, Ahn et al. [36] proposed that the anterolateral approach tended to result in wider entrance and lower failure load compared with anteromedial approach. Besides, the outlet of the OTLT needed to be located at the inferior lateral aspect of the PCL anatomic footprint. It may lead to a fairly short tunnel if the anterolateral approach is used and may affect the fixation of the graft. Certainly, further biomechanical studies are needed to verify this hypothesis. Third, the patients included were from a single ethnicity of Chinese Han nationality. Therefore, certain limitations may exist when the reference data were used for other races. Lastly, as the current study was based on 2D CT images and 3D knee models, future cadaveric, biomechanical, and clinical studies are nonetheless needed to validate our findings and figure out whether the tangential low tibial‐tunnel is still optimal at the levels of biomechanics and clinical results.

### 4.5 Prospects of Clinical Application

To build an anteromedial OTLT for arthroscopic transtibial PCL reconstruction, this study suggested to locate the PCL drilling guide pin 62 mm below the tibial plateau and 15 mm medial to the tibial crest with an angle of 48°, toward the exit point 30 mm below the tibial plateau and inferior lateral aspect of the PCL anatomic insertion site. In addition, we should control the depth of 46 mm when drilling the guide pin and reamer to reduce the risks of neurovascular injury. While the values should be moderately adjusted according to gender and height, and corresponding differences need to be added on when planning to conduct an OTLT based on 2D CT images relative to 3D knee models. As this was a retrospective CT image study, the parameter values of the OTLT may generate certain variations when applied clinically. Therefore, future cadaveric, biomechanical, and clinical studies are still needed to further verify our findings.

## 5 Conclusion

Based on 2D CT images and 3D knee models, the OTLT could be created and relevant parameters were measured. These quantitative parameter values could be used as reference data for orthopedic surgeons to build an anteromedial OTLT during the arthroscopic transtibial PCL reconstruction.

## Author Contributions

**Laiwei Guo, Xiaoyun Sheng**, and **Caijuan Dai:** performed the research, interpreted data, drafted and wrote the manuscript. **Zhongcheng Liu, Xingwen Wang, Lianggong Zhao, Xiaohui Zhang, Rui Bai**, and **Xiaoli Zheng:** collected and analyzed the data. **Bin Geng** and **Meng Wu:** supervised the data collection. **Yuanjun Teng** and **Yayi Xia:** reviewed the paper and revised the manuscript.

## Ethics Statement

Approval was obtained from the ethics committee of Lanzhou University Second Hospital (No. 2022A‐556).

## Conflicts of Interest

The authors declare no conflicts of interest.
